# WeBIAS: a web server for publishing bioinformatics applications

**DOI:** 10.1186/s13104-015-1622-x

**Published:** 2015-11-02

**Authors:** Paweł Daniluk, Bartek Wilczyński, Bogdan Lesyng

**Affiliations:** Bioinformatics Laboratory, Mossakowski Medical Research Centre, Pawińskiego 5, 02-106 Warsaw, Poland; Department of Biophysics, Faculty of Physics, University of Warsaw, Warsaw, Poland; Institute of Informatics, University of Warsaw, Warsaw, Poland

**Keywords:** Bioinformatics, Programming, Software engineering, Web services

## Abstract

**Background:**

One of the requirements for a successful scientific tool is its availability. Developing a functional web service, however, is usually considered a mundane and ungratifying task, and quite often neglected. When publishing bioinformatic applications, such attitude puts additional burden on the reviewers who have to cope with poorly designed interfaces in order to assess quality of presented methods, as well as impairs actual usefulness to the scientific community at large.

**Results:**

In this note we present WeBIAS—a simple, self-contained solution to make command-line programs accessible through web forms. It comprises a web portal capable of serving several applications and backend schedulers which carry out computations. The server handles user registration and authentication, stores queries and results, and provides a convenient administrator interface. WeBIAS is implemented in Python and available under GNU Affero General Public License. It has been developed and tested on GNU/Linux compatible platforms covering a vast majority of operational WWW servers. Since it is written in pure Python, it should be easy to deploy also on all other platforms supporting Python (e.g. Windows, Mac OS X). Documentation and source code, as well as a demonstration site are available at http://bioinfo.imdik.pan.pl/webias.

**Conclusions:**

WeBIAS has been designed specifically with ease of installation and deployment of services in mind. Setting up a simple application requires minimal effort, yet it is possible to create visually appealing, feature-rich interfaces for query submission and presentation of results.

## Background

Our experience shows that one of the most ungratifying tasks when developing a novel computational method is preparing a publicly accessible server. Only the most prominent research groups have resources to maintain an integrated web portal with such extended functionalities. Although there exist excellent Web Services frameworks like Soaplab [1] or Opal Toolkit [2], which were designed to provide a machine access to services via protocols like WSDL, they require significant installation effort and expert knowledge, and are most useful in case of popular, high-demand applications. They are, however, less attractive when taking into account user experience, lacking features like user registration, easy retrieval of old results or customized presentation of results.

More advanced environments for work-flow design and visualisation like Galaxy [3] and Taverna [4] have vibrant communities, but require significant effort from newcomers who would like either to publish their own services or use existing ones.

In turn, the GMOD (Generic Model Organism Database) project has developed a framework based on the Drupal CMS [5]. It is well suited for developing services requiring an efficient database backend, but it has several dependencies which make its installation and setup difficult.

Néron et al. in their article presenting Mobyle framework [6] propose several concepts for a bioinformatics server such as homogeneous user interfaces to heterogeneous programs, persistent user workspaces, XML description of interfaces and a network-enabled tools. Their web framework—Mobyle—has been designed with these in mind. It can be used to host a large number of services, and establish a network of servers which may forward requests to other Mobyle sites. This set of features is particularly useful for creating a large bioinformatics hub with various services and workflows available to users. It is inevitable, that such functionality comes at a price of the overall complexity.

Web BioInformatics Application Server (WeBIAS) has been designed to fill the need for easily configurable, feature rich bioinformatics portals. It makes it possible to create a rudimentary version of a new service starting from scratch in less than an hour. It is based on an advanced template engine allowing unlimited possibilities of rendering computed results including embedding rich media content. It also fulfills all concepts proposed by Néron et al., except cooperation between multiple sites.

Most, if not all, scientific computation tools can be packaged as standalone programs. WeBIAS provides infrastructure to wrap such programs with a web GUI, facilitate job submission and scheduling, and present computation results whenever they are ready. In principle any command line program or script can be used. It is only required that it returns results in an XML format.^1^ It may also generate several output files (e.g. images) which are automatically stored. Since computations may require significant resources and time, and may have to be scheduled for later execution, WeBIAS handles job scheduling by itself or may use external schedulers like Torque/PBS or SLURM. Computations can be carried out on any number of servers.

### Architecture

**Fig. 1:**
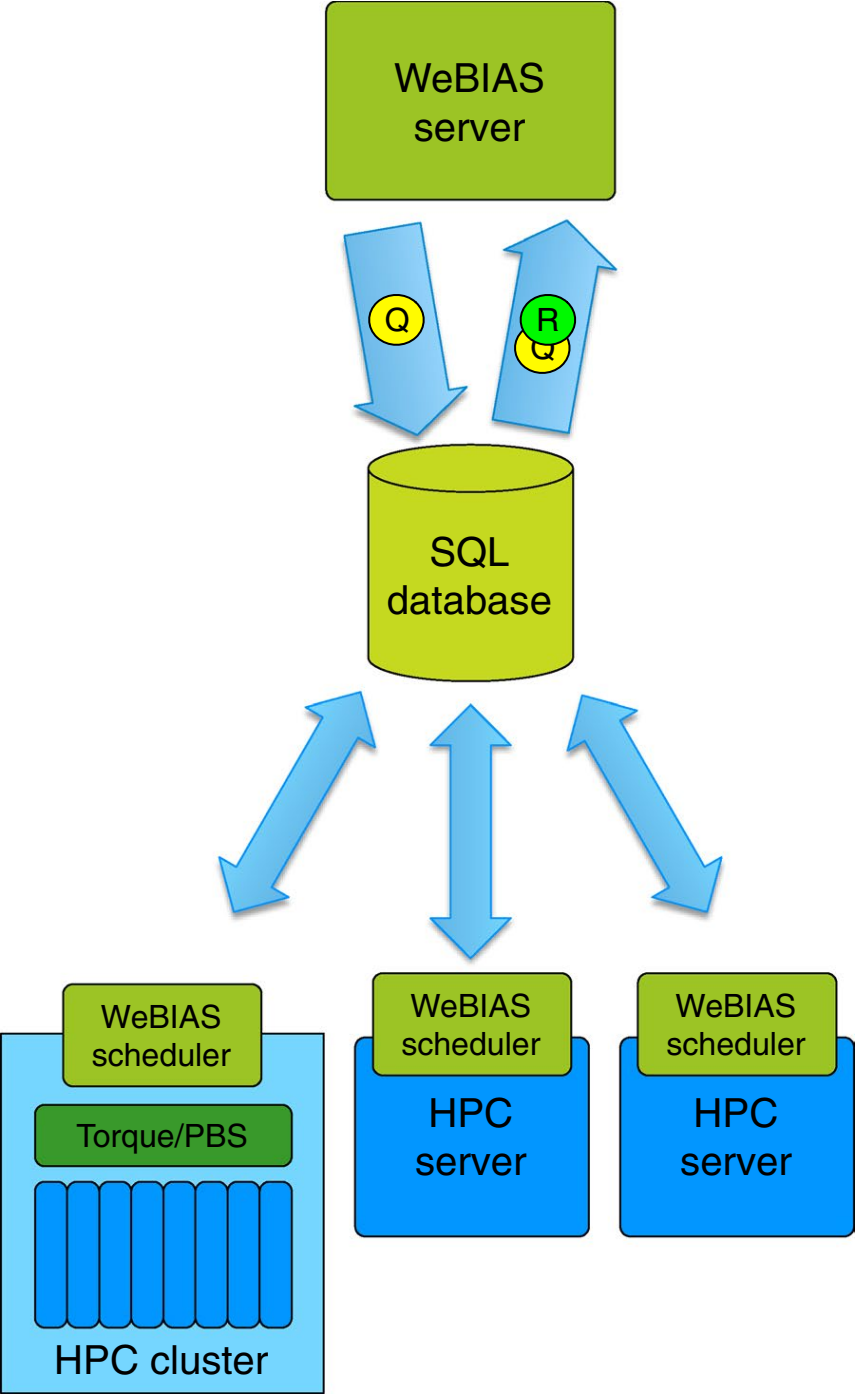
WeBIAS architecture. WeBIAS comprises a web server and one or more schedulers. Web server provides a user interface allowing query submission and result presentation. Queries and results are stored in the SQL database. Schedulers grab unprocessed queries, oversee computations and store results in the database

WeBIAS comprises two main components: a web server front end and a computational backend (see Fig. 1). The front end is responsible for receiving queries and presentation of results. It also provides user interface for all features discussed below. The backend schedules, runs and supervises programs which perform computations.

In most cases it is infeasible to perform computations on the machine running a web server for performance and security reasons. Computations may require access to resources which should not be stored on the most exposed machines. We presume that most research groups have access to HPC servers, which could be used to process queries collected by a web service. However, bridging a gap between a web server exposed to the internet and HPC machines without compromising security is difficult to be done in an ad-hoc manner. In WeBIAS, the web front end communicates with its computational backends only through an SQL database. All queries accepted by the web server are stored in the database. Backend instances periodically check for unserviced queries and schedule them for computation. This separation guarantees that even if the computer running the front end gets compromised, there is no practical way to gain control of the computational environment by ordering execution of arbitrary tasks.^2^

Most applications in addition to numerical results produce several output files (e.g. images, biological sequences or structures, large tables etc.). WeBIAS uses its database to store these. Computational backend loads all result files into the database, and the frond end serves them to users. This solution is much better than using a file system shared between computing environment and a web server, or using separate file systems with a file replication scheme. The latter solution is a potential security breach, and in several cases might not even be technically feasible.

WeBIAS allows running several backend instances concurrently. Each instance may be configured to utilize different computational resources (e.g. clusters, job queues, etc.) and perform computations for a subset of applications. Such flexibility enables optimal utilization of limited resources and does not require exclusive allocation of servers. This is particularly important for smaller research groups which own a limited number of servers or use shared infrastructure.

Although these features make WeBIAS useful in complex environments, it can be very easily deployed on a single server. Minimalistic configuration requires preparing only a single configuration file, and starting two programs (front- and backend).

## Methods

WeBIAS is written in Python and uses the CherryPy engine [7]. The web front end is a standalone web server, which can be run independently or integrated into existing web infrastructure. All data is stored in the relational SQL database which is accessed using SQLAlchemy object-relational mapping framework [8].

The backend is run independently from the web sever component, and communicates with it only via the SQL database. It periodically checks for unserviced queries, and runs appropriate computations either directly or using a job scheduling system.^3^ It also monitors running jobs, and stores results in the database when they finish. Several instances of a backend component may be run concurrently. If more than one instance is capable of handling a particular job, they may compete for it. SQL locking mechanisms are used to avoid eventual deadlocks and race conditions.

**Fig. 2:**
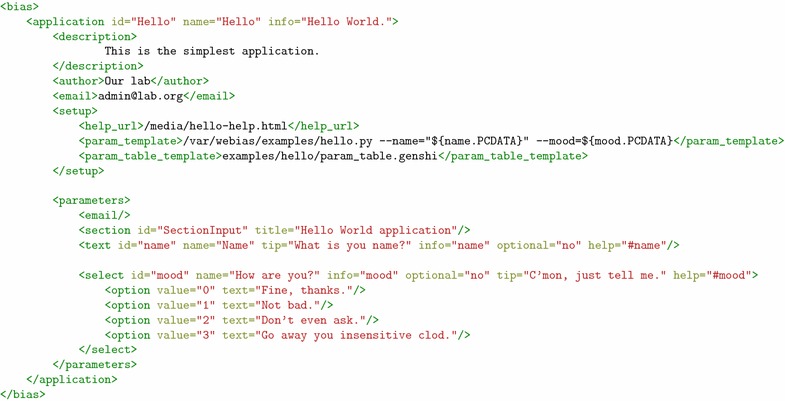
Sample XML definition of an application. Applications are described using XML. Such file contains generic information like application name, author, short description, etc. The <setup> section contains a template for converting input data into a command line of a program to be called, and custom template files. The <parameters> section contains definition of fields to be displayed in the job submission form

Our goal was to design a server which would provide a rich user experience without requiring significant effort from application developers. There are two basic application dependent functionalities which have to be customized: job submission form and result presentation. Sophisticated forms can be designed by providing an XML definition containing descriptions of fields and their arrangement. Definition of a simple application is presented in Fig. 2.

At present WeBIAS supports single value fields of common types (integer, float, boolean, string), as well as parameter groups with definable semantics (e.g. containing parameters which are mutually exclusive). Dynamic forms with variable number of parameters are also supported with a client side Javascript. Input is validated before actual submission takes place to allow easy corrections without relying on the browser “back button” feature. WeBIAS also offers specialized fields for input of bioinformatics data by either giving a database accession code or uploading a file. Currently it links to public databases of molecular structures such as PDB and SCOP, and provides verification of files in PDB format. These features depend on the Biopython toolset [9]. Gnosis Utils [10] are used to convert XML definitions to Python objects. Thus an application description is automatically converted to a hierarchy of objects which contains objects belonging to classes corresponding to field types with all relevant methods for field behavior and validation. In order to extend WeBIAS to support a new parameter type, it is enough to subclass an existing field type. This will automatically extend the set of XML tags allowed in application definition.

Custom field types can be used to provide special input validation and/or preprocessing. This is, for example, the case of bioinformatics data fields, where a database accession code is converted to an actual data file. Similarly, one can implement a field which converts various file formats to the one required by an application or checks whether a supplied file has particular features required for computation. This approach has an advantage over postponing such operations until the job is being processed, because a user can be interactively informed about problems in his submission.

**Fig. 3:**
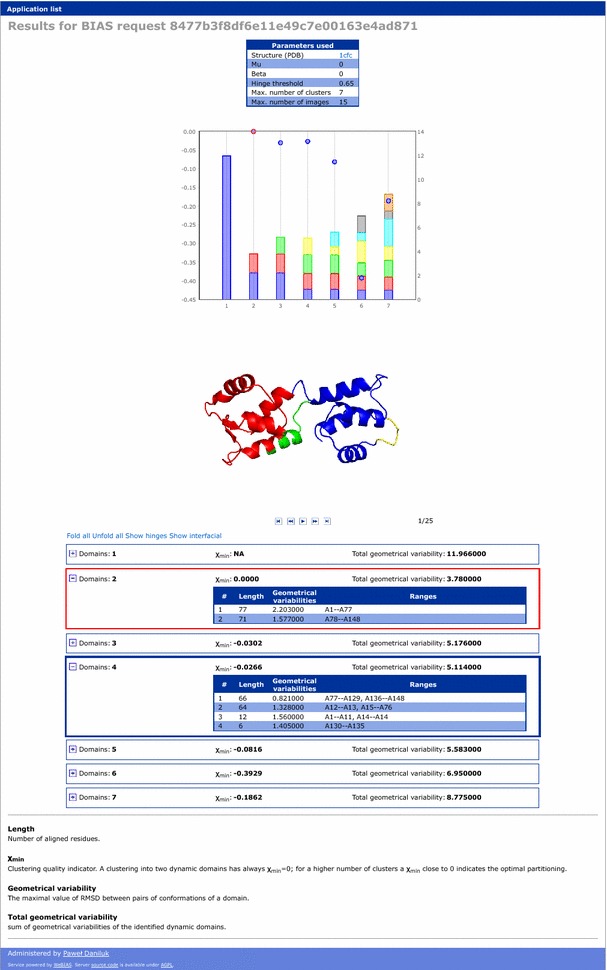
Example of a result presentation. Each application in WeBIAS may have a template for presenting results, which are stored internally in XML format. Templates are used to convert results to HTML. They may have dynamic elements programmed in JavaScript. Here the chart is rendered by client-side routines and is interactive, molecular renderings are dynamically downloaded, and table elements are collapsible

Clear and visually pleasing presentation of results may not correlate with scientific value of a bioinformatic tool. Nevertheless, it may have a significant impact on its popularity among users, and thus contribute to a success of a tool or an underlying publication. In many cases, however, rapid deployment is of greater value than a sophisticated interface. WeBIAS addresses these contradicting demands through separation of computed results, which are stored in XML format, from their presentation. The conversion of the former into HTML content, which is rendered in user’s web browser, is done using a template engine (Genshi [11]). An example of a dynamic webpage presenting results in shown in Fig. 3. A template may be modified at any time, and such update will apply to all already computed results. If a template is not supplied, WeBIAS falls back to a default template which presents data in a tabular layout. Default behavior comprises a set of rules which preserve the treelike structure of an XML encoding of a result. Default templates may be used for testing, and also whenever instant deployment of an application is required.

There are several security considerations, which should be taken into account when designing such a web server. Potential eavesdropping and man-in-the-middle attacks are prevented by using SSL encryption. WeBIAS uses CherryPy SSL features or, if it is installed behind a proxy server which handles encryption, it discerns safe encrypted connections from unencrypted ones by custom headers introduced into HTTP requests by a proxy server. WeBIAS manages its own user authentication and authorization. Anyone may use publicly available services without providing any identification. This is usually a requirement in case of services, which are peer reviewed in order to preserve anonymity of reviewers.^4^ Users may also provide an e-mail address to receive job status updates, or register to gain access to its full functionality. Logins and passwords are stored in the SQL database. Before a new account is enabled, verification of the e-mail address is performed. Also, it is impossible to submit a job with an e-mail of a registered user without being logged in. These measures were introduced to prevent maliciously inducing WeBIAS to send unsolicited e-mails. All requests are stored on the server for future perusal. Requests submitted anonymously are publicly available to everyone knowing an UUID. This applies also to requests submitted with an e-mail address by unregistered users. However, after registration all requests, including those made before registration, become password protected. Therefore, registered users can reasonably expect that their queries and computation results remain private. On the other hand, queries submitted anonymously can be shared by public at large.

For larger installations, where several authors may publish their services on the portal, there is an option of promoting certain users to the power user role to give them administrative access to their applications and schedulers. Power users may be granted rights to access yet unpublished applications in order to perform tests in production environment before actual publication, enable/disable applications, view submitted requests and schedule reports on application health and activity. These rights are independently set for each application to allow access to people’s own services without compromising security of others. In this manner developers may be allowed to test their applications in the production environment, and group members or collaborators may gain access to a yet unpublished service.

All files (supplied by users as parameters, and computed) are stored in the database. Backend instances are responsible for downloading proper files to a temporary directory where a job is executed and for uploading resulting files. In order to minimize security risks of unnecessarily exposing vulnerable files (e.g. due to cunningly formed URL requests^5^) no data is stored in the file system and no file system files are served.

WeBIAS has been designed in a manner allowing deployment without compromising network security. The web front end may and should be ran on a server placed in a so-called perimeter network (or DMZ). Backends responsible for job scheduling may be put on the internal network with all privileges required for job submission. All communication between these components is performed *via* SQL database and comprises only contents of submitted requests rendered in XML format. Before a request is submitted it is validated by the front end. Each form field is checked according to its type and allowed values. It is also possible to include validators for specific file formats. Such architectural solution helps to mitigate most attack scenarios. Compromising a front end does not give an attacker a direct method to take over computational resources. At best he would be able to inject crafted queries into the database. These would have to defeat sanity checks imposed by a backend and cause it to malfunction or cause an application to fail. Having to penetrate these layers one by one makes an attack difficult. Communication through a database eliminates the possibility of exploiting eventual flaws (e.g. buffer overflows) in the implementation of a communication protocol. We can envision only one attack vector which cannot be prevented by this architecture—when validated input would cause a fault in the application program, which would in turn compromise the system. This, however, may be mitigated by running computations with a low privilege.

## Discussion

Providing public access to computational methods is instrumental to their dissemination. Although in most cases making binaries available for download is considered sufficient, many users do not wish to expend their effort on going through the hassle of trying out such programs. On the other hand, web interfaces are familiar to everyone, do not require any installation and allow to try an application out almost effortlessly. In most cases, however, authors of computational software lack initiative to develop sophisticated web sites or to learn complex web service frameworks. Also development costs of feature rich in-house solutions can hardly be justified for most research groups. Nevertheless, they usually have a usable application, which can be easily adapted for WeBIAS.

WeBIAS has been developed to close the gap, and allow for a quick and smooth publication of computational services. A simple service can be made available very rapidly using default templates. Experienced users may use WeBIAS as a framework for the development of advanced applications. In both cases WeBIAS provides all routines required for query submission, job scheduling and result retrieval. WeBIAS may be installed on a single computer to achieve a self contained solution for services requiring low computing power, or deployed in an extensive computational environment with diverse computing resources.

WeBIAS has been used to set up an *Essentia Proteomica* portal [12] with services for structural alignment of proteins [13] and detection of dynamic domains [14]. Several other services were made available with its use [15, 16].

## Availability and requirements

- Project name: WeBIAS
- Project home page: http://bioinfo.imdik.pan.pl/webias
- Operating system(s): Platform independent
- Programming language: Python, JavaScript
- Other requirements: MySQL server, CherryPy 3.8 or higher, SQLAlchemy 0.8 or higher, Genshi 0.6 or higher
- License: GNU AGPL
- Any restrictions to use by non-academics: None

## Abbreviations

AGPL: Affero General Public License
CMS: Content Management System
HPC: High Performance Computing
HTML: HyperText Markup Language
SQL: Structured Query Language
SSL: Secure Sockets Layer
UUID: Universally Unique IDentifier
WSDL: Web Services Description Language
XML: eXtensible Markup Language

## Footnotes

1 In case of programs which cannot or should not be modified, this can be easily achieved with an additional wrapper script.

2 Unless programs used for computations are badly designed and do not sanitize their parameters properly.

3 Presently Torque/PBS and SLURM are supported. Support for other queuing systems may be easily added by providing methods for queuing a job and checking whether it has finished.

4 However, one always has to bear in mind, that connection origin may sometimes reveal identity, if the number of persons potentially involved is limited.

5 A naïve attack of this type might involve requesting http://myserver.com/site/directories/files/../../../../etc/passwd.

## References

[1] Senger M, Rice P, Bleasby A, Oinn T, Uludag M. Soaplab2: more reliable Sesame door to bioinformatics programs. In: Bioinformatics Open Source Conference, BOSC, vol 8. 2008.

[2] Ren J, Williams N, Clementi L, Krishnan S, Li WW (2010). Opal web services for biomedical applications. Nucleic Acids Res..

[3] Giardine B, Riemer C, Hardison RC, Burhans R, Elnitski L, Shah P, Zhang Y, Blankenberg D, Albert I, Taylor J (2005). Galaxy: a platform for interactive large-scale genome analysis. Genome Res..

[4] Oinn T, Addis M, Ferris J, Marvin D, Senger M, Greenwood M, Carver T, Glover K, Pocock MR, Wipat A (2004). Taverna: a tool for the composition and enactment of bioinformatics workflows. Bioinformatics..

[5] Papanicolaou A, Heckel DG (2010). The GMOD Drupal Bioinformatic Server Framework. Bioinformatics..

[6] Néron B, Ménager H, Maufrais C, Joly N, Maupetit J, Letort S, Carrere S, Tuffery P, Letondal C (2009). Mobyle: a new full web bioinformatics framework. Bioinformatics..

[7] Delon R, Brewer R, Hellegouarch S, Wyglendowski C, Wecker L. CherryPy 3.6: A Minimalist Python Web Framework. 2015. http://cherrypy.org. Accessed 2015.

[8] Bayer M. SQLAlchemy: The Python SQL Toolkit and Object Relational Mapper. http://www.sqlalchemy.org. Accessed 30 Oct 2015.

[9] Cock PJA, Antao T, Chang JT, Chapman BA, Cox CJ, Dalke A, Friedberg I, Hamelryck T, Kauff F, Wilczynski B, de Hoon MJL (2009). Biopython: freely available Python tools for computational molecular biology and bioinformatics. Bioinformatics..

[10] Mertz D, McIngvale F. Gnosis Utils 1.2.2. http://www.gnosis.cx/download. Accessed 30 Oct 2015.

[11] Thomas A, Lenz C, Borgström J, Good M, Cross S. Genshi 0.6: Python Toolkit for Generation of Output for the Web. http://genshi.edgewall.org. Accessed 30 Oct 2015.

[12] Daniluk P. Essentia Proteomica. http://dworkowa.imdik.pan.pl/EP. Accessed 30 Oct 2015.

[13] Daniluk P, Lesyng B (2011). A novel method to compare protein structures using local descriptors. BMC Bioinform..

[14] Dziubiński M, Daniluk P, Lesyng B. ResiCon: a method for the identification of dynamic domains, hinges and interfacial regions in proteins. Bioinformatics. 2015. 10.1093/bioinformatics/btv525. 10.1093/bioinformatics/btv525 26342233

[15] Wilczynski B, Darzynkiewicz M, Tiuryn J. MEMOFinder: combining de novo motif prediction methods with a database of known motifs. Nature Precedings. 2008. Available from http://hdl.handle.net/10101/npre.2008.2289.2.

[16] Wilczyński B, Dojer N (2009). BNFinder: exact and efficient method for learning Bayesian networks. Bioinformatics..

